# Current treatment patterns within 1 year after prostate cancer diagnosis in Korean patients over 75 years old: a retrospective multicenter study

**DOI:** 10.1016/j.prnil.2022.08.003

**Published:** 2022-08-29

**Authors:** Dong Jin Park, Ho Won Kang, Se Yun Kwon, Young Jin Seo, Kyung Seop Lee, Byung Hoon Kim, Teak Jun Shin, Won Tae Kim, Yong-June Kim, Seok Joong Yun, Sang-Cheol Lee, Jae-Wook Chung, Seock Hwan Choi, Jun Nyung Lee, Hyun Tae Kim, Tae-Hwan Kim, Eun Sang Yoo, Tae Gyun Kwon, Wonho Jung, Yun-Sok Ha

**Affiliations:** 1 Department of Urology, Dongguk University College of Medicine, Gyeongju, Korea; 2 Department of Urology, Chungbuk National University Hospital, College of Medicine, Chungbuk National University, Cheongju, Korea; 3 Department of Urology, Keimyung University Gyeongju Dongsan Hospital, Keimyung University School of Medicine, Gyeongju, Korea; 4 Department of Urology, Keimyung University Dongsan Hospital, Keimyung University School of Medicine, Daegu, Korea; 5 Department of Urology, School of Medicine, Kyungpook National University, Daegu, Korea

**Keywords:** Aging, Prostate Cancer, Treatment

## Abstract

**Background:**

We aimed to evaluate the current status of first-line treatment options for prostate cancer in patients aged ≥75 years in Korea.

**Materials and methods:**

The study included 873 patients diagnosed with biopsy-proven prostate cancer at 5 institutions in Korea from January 2009 to December 2018. Inclusion criteria were aged ≥75 years at diagnosis, prostate biopsy with ≥12 cores, and follow-up period ≥1 year. Clinical data were retrospectively collected from electronic medical records.

**Results:**

Primary treatment for prostate cancer in patients aged ≥75 years included androgen deprivation therapy (ADT) (n = 614), radical prostatectomy (RP) (n = 114), and radiation therapy (n = 62). Among patients with RP, nine patients received ADT before RP. The RP group was younger with better Eastern Cooperative Oncology Group Performance Status (ECOG PS), lower initial prostate-specific antigen (PSA), Gleason score (GS), max percent positive cores, less positive cores, and less advanced clinical Tumor Node Metastasis (TNM) stage compared with the ADT group. Multivariate analysis showed that age, ECOG PS, and PSA were independent prognostic factors for RP. When the ADT group was classified by therapeutic regimens, the most common therapeutic regimen was maximal androgen blockade (MAB) (n = 571), and leuprolide + bicalutamide (n = 330) was the most common MAB regimen. Multivariate analysis for secondary treatment showed that age, ECOG PS, GS, and clinical N1 or M1 stage were independent predictive factors. Enzalutamide was the most preferred treatment for tertiary treatment.

**Conclusion:**

In patients with prostate cancer aged ≥75 years, the most common treatment option was MAB, and the leuprolide + bicalutamide was the most common MAB regimen. Age, ECOG PS, and PSA are the useful indicators of surgical treatment, which increased during the study period. Younger patients with high GS and advanced clinical stage were more likely to undergo secondary treatment.

## 1 Introduction

The increased proportion of people over 65 years indicates societal aging.^1^ In Korea, which became an “aged society” in 2018, the proportion of the population over 65 was 15.8% in 2020.^1^^,^^2^ According to the complete life tables in the Korean Statistical Information Service, the life expectancy of Korean males is persistently increasing and was 80.5 years old in 2020.^3^

In Korea, prostate cancer is one of the most common cancers in men aged 65 years and over, and the incidence of prostate cancer is increasing in recent years.^4^ The incidence of prostate cancer peaks at 75–79 years. Men aged 65 years and over account for 77.2% of prostate cases, and men aged 75 years and over account for 35.9% of prostate cases.^4^^,^^5^ In the National Comprehensive Cancer Network guidelines for prostate cancer, estimated life expectancy and risk stratification are important factors determining treatment modalities^6^, which include radical prostatectomy (RP) in patients with a life expectancy of 10 years or more.^6^

Several studies focused on RP in elderly patients.^7^, ^8^, ^9^, ^10^, ^11^ An observational study using the National Prostate Cancer Register of Sweden reported that the proportion of patients over 70 years old with localized prostate cancer who underwent RP was lower than the proportion of younger patients who underwent RP; however, an increasing proportion of patients undergoing RP was observed during the study period.^7^ Two studies that investigated treatment patterns of Korean patients with prostate cancer reported similar results in patients aged 75 years or older.^8^^,^^9^ A retrospective study of Korean patients with prostate cancer reported that RP was an appropriate treatment option for the selected patients aged 75 years or older.^10^ Another study using National Health Insurance Sharing Service data reported that the patients aged 75 years or older who underwent robot-assisted RP (RARP) for non-metastatic prostate cancer had similar survival rates as patients who underwent radiation therapy (RT).^11^

In Korea, prostate cancer more rapidly increased from 2015 to 2019 than from 2009 to 2015.^4^ However, most previous studies did not include patients recently diagnosed with prostate cancer from 2015 to 2019.^8^, ^9^, ^10^ Moreover, these studies, except the one that did not include clinical data from prostate cancer^8^^,^^9^^,^^11^, included clinical data investigated for only RP as the primary treatment.^10^ To investigate more recent treatment patterns, including clinical data for prostate cancer, we aimed to evaluate the current status of treatment options for prostate cancer in patients aged ≥75 years in Korea.

## 2 Materials and methods

### 2.1 Ethics statement

This retrospective study was performed with the approval of the Institutional Review Board of Dongguk University Gyeongju Hospital (IRB number: 110757-202206-HR-02-02). The study was conducted in accordance with the relevant laws and regulations, good clinical practices, and ethical principles, as described in the Declaration of Helsinki. Informed consent was waived by the board due to the retrospective nature of the study.

### 2.2 Patients

Clinical data were retrospectively collected from electronic medical records of patients diagnosed with prostate cancer at five institutions from January 2009 to December 2019. Inclusion criteria were (i) 75 years or older at time of diagnosis; (ii) diagnosis of prostate cancer with prostate biopsy of 12 or more cores; (iii) prostate cancer with known clinical TNM stage and Gleason score (GS); (iv) follow-up period of more than one year. Of the 881 patients who satisfied the inclusion criteria, seven patients with unknown clinical TNM stage and one patient with an unknown GS were excluded. Thus, 873 patients were enrolled in the study.

Patients were divided according to the primary treatment into androgen deprivation therapy (ADT) (n = 614), RP (n = 114), and RT (n = 62) groups. We analyzed the proportions of patients who received hormonal therapy regimens and maximal androgen blockade (MAB) regimens. We compared the baseline and clinicopathological characteristics of patients who underwent ADP or RP as the primary treatment. We also analyzed the baseline and clinicopathological characteristics in patients who received secondary and tertiary treatments. We compared the baseline and clinicopathological characteristics of patients as well as primary treatment patterns based on the year of diagnosis (2009–2014 vs. 2015–2019), age of diagnosis (75–79 vs. 80 years or older), and clinical stage (localized prostate cancer vs. locally advanced and metastatic prostate cancer).

### 2.3 Statistical analysis

The Student's *t*-test or Mann–Whitney test was used for continuous variables. The Chi-square test or Fischer Exact test was used for categorical variables. Multivariate Cox regression analyses were performed to determine the predicting factors for undergoing RP and secondary treatment. The odds ratio (OR) and 95% confidence interval were determined. All statistical analyses were performed using the Statistical Package for the Social Sciences, version 27.0 (SPSS Inc., Chicago, IL, USA), and *P* values < 0.05 were considered statistically significant.

## 3 Results

We retrospectively reviewed the medical records of 873 patients diagnosed with prostate cancer at five institutions from January 2009 to December 2019. The median age was 78.0 (75.0–94.0) years, the median prostate-specific antigen (PSA) was 18.6 (0.6–6520.0) ng/ml, and the median prostate volume was 37.1 (6.5–224.6) ml. GSs were ≥8 in 57.4% of patients, and 19.2% of patients had metastatic prostate cancer (Table 1).

**Table 1:** Baseline and clinicopathological characteristics of the study population

Characteristics	Patients (n = 873)
Age, years, median (range)	78.0 (75.0–94.0)
BMI, kg/m^2^, mean ± SD	23.2 ± 2.9
ECOG PS, n (%)	
0	481 (55.2)
1	250 (28.7)
2	137 (15.7)
3	3 (0.3)
Hypertension, n (%)	397 (45.5)
Diabetes mellitus, n (%)	180 (20.6)
Other medical histories, n (%)	
Cardiac disease	91 (10.4)
Nephrotic disease	21 (2.4)
Pulmonary disease	46 (5.3)
Brain disease	60 (6.9)
Other cancer	85 (9.7)
Initial PSA, ng/ml, median (range)	18.6 (0.6–6520.0)
Prostate volume, cc, median (range)	37.1 (6.5–224.6)
Gleason score, n (%)	
≤6	123 (14.1)
7	249 (28.5)
8	275 (31.5)
9	176 (20.1)
10	50 (5.7)
Number of positive cores, median (range)	6.0 (1.0–30.0)
Max percent of positive core, median (range)	80.0 (1.0–100.0)
Clinical T stage, n (%)	
≤T2	489 (56.0)
≥T3	384 (44.0)
Clinical N stage, n (%)	
N0	698 (80.0)
N1	175 (20.0)
Clinical M stage, n (%)	
M0	705 (80.8)
M1	168 (19.2)

BMI, body mass index; ECOG PS, Eastern Cooperative Oncology Group Performance Status; PSA, prostate-specific antigen; T, tumor; N, lymph nodes; M, metastasis.

**Fig. 1:**
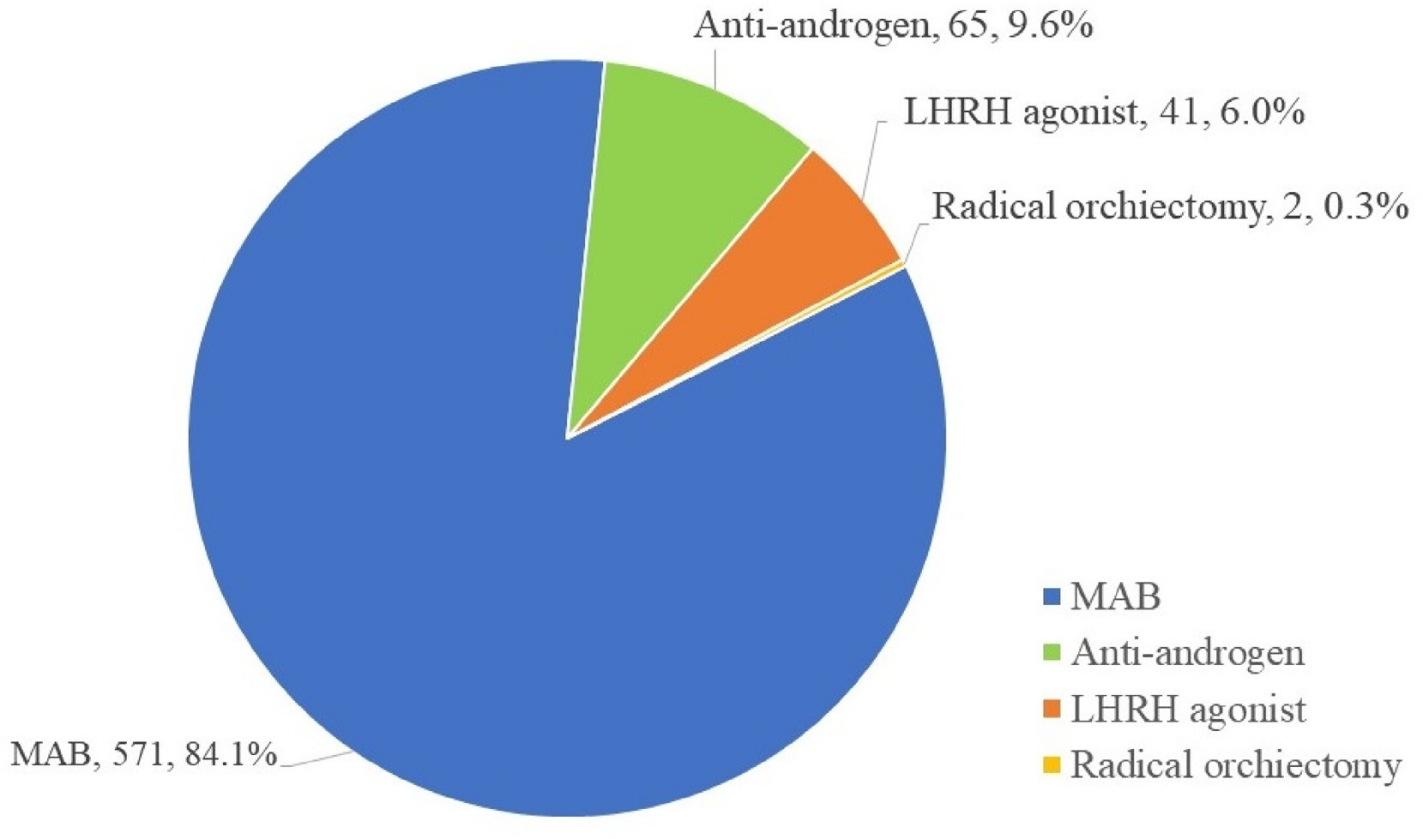
The proportions of hormone therapy regimens.

**Fig. 2:**
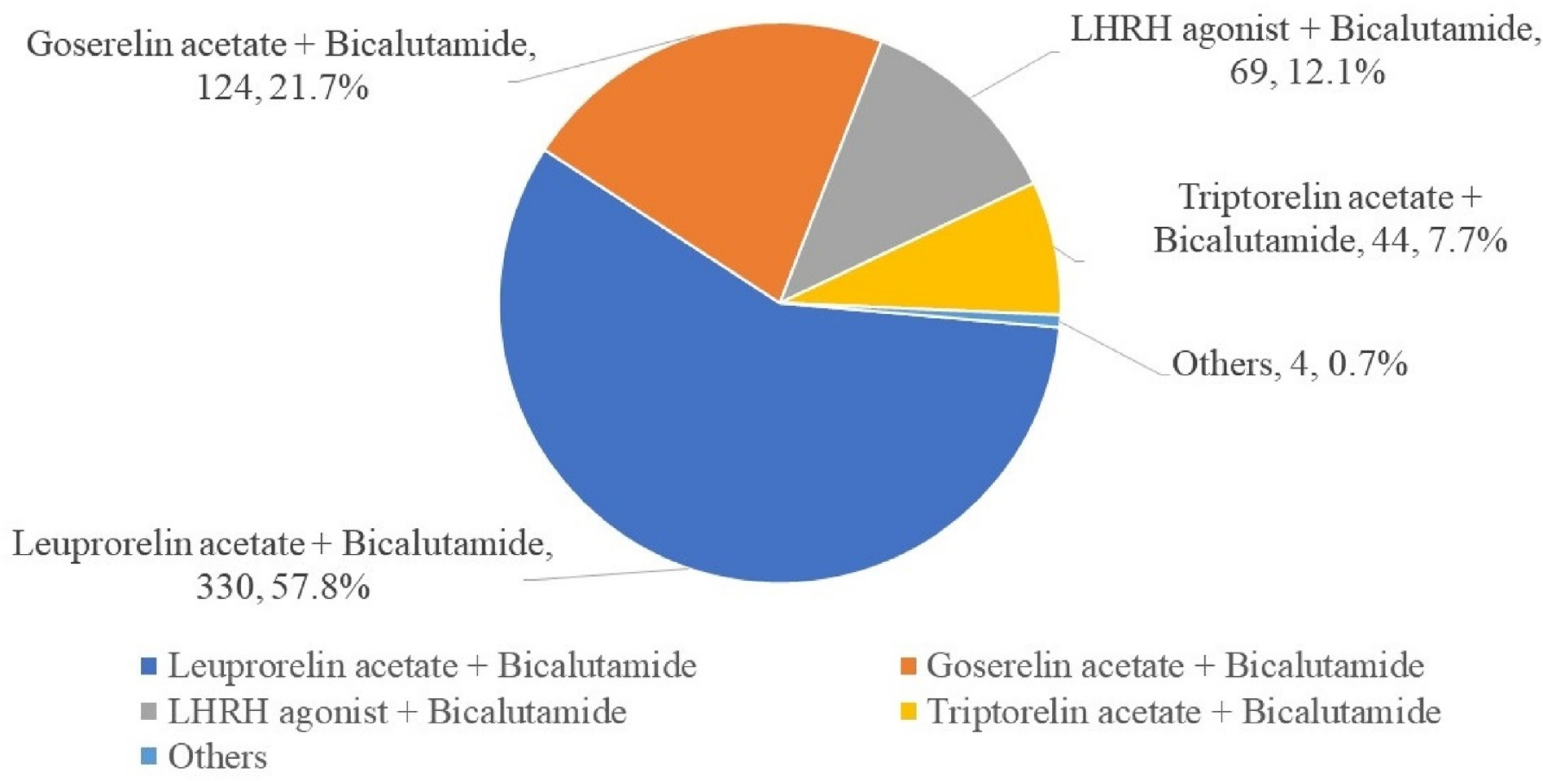
The proportions of maximal androgen blockade regimens by drug ingredient.

Patients were divided according to the primary treatment into ADT (n = 614), RP (n = 114), and RT (n = 62) groups. The most common therapeutic regimen in the ADT group was MAB (84.1%) (Fig. 1). Among patients receiving MAB, leuprorelin acetate + bicalutamide (57.8%) was the most common regimen (Fig. 2).

The RP group was younger (76.0 vs. 79.0, *P* < 0.001) and had a higher body mass index (23.8 ± 3.0 vs. 23.0 ± 2.7, *P* = 0.004) compared with age and body mass index in the ADT group. In addition, the Eastern Cooperative Oncology Group Performance Status (ECOG PS), initial median PSA (7.9 vs. 26.8 ng/ml, *P* < 0.001), GS, median number of positive cores, median max percent of positive cores, and advanced clinical TNM stage (*P* < 0.001) were lower in the RP group than these parameters in the ADT group (Table 2). Among the RP group, 7.9% of patients underwent neoadjuvant hormone treatment and 4.4% of patients underwent adjuvant hormone treatment.

**Table 2:** Comparisons of baseline and clinicopathological characteristics between ADT and RP groups as the primary treatment

	ADT (n = 614)	RP (n = 114)	*P*-value
Age, years	79.0 (75.0–94.0)	76.0 (75.0–82.0)	<0.001
BMI, kg/m^2^	23.0 ± 2.7	23.8 ± 3.0	0.004
ECOG PS			<0.001
0	281 (45.8)	106 (93.0)	
≥1	332 (54.1)	8 (7.0)	
Hypertension	279 (45.6)	60 (52.6)	0.171
Diabetes mellitus	125 (20.4)	26 (22.8)	0.571
Initial PSA, ng/ml	26.8 (1.8–6520.0)	7.9 (2.7–46.0)	<0.001
Total prostate volume, cc	37.9 (6.5–224.6)	36.0 (13.0–124.0)	0.190
Gleason score			<0.001
≤6	72 (11.7)	26 (22.8)	
7	154 (25.1)	48 (42.1)	
≥8	388 (63.2)	40 (35.1)	
Number of positive cores	7.0 (1.0–30.0)	4.0 (1.0–13.0)	<0.001
Max percent of positive core, %	87.5 (5.0–100.0)	50.0 (1.0–100.0)	<0.001
Clinical T stage			<0.001
≤T2	306 (49.8)	90 (78.9)	
≥T3	308 (50.2)	24 (21.1)	
Clinical N stage			<0.001
N0	458 (74.6)	111 (97.4)	
N1	156 (25.4)	3 (2.6)	
Clinical M stage			<0.001
M0	457 (74.4)	113 (99.1)	
M1	157 (25.6)	1 (0.9)	

ADT, androgen deprivation therapy; BMI, body mass index; ECOG PS, Eastern Cooperative Oncology Group Performance Status; M, metastasis; N, lymph nodes; PSA, prostate-specific antigen; RP, radical prostatectomy; T, tumor.

According to the multivariable logistic regression analysis, the predicting factors for undergoing RP as the primary treatment were age (OR 0.653, *P* < 0.001), ECOG PS ≥ 1 (OR 0.066, *P* < 0.001), and serum PSA level (OR 0.943, *P* < 0.001). No other factors were associated with RP as the primary treatment (Table 3).

**Table 3:** Analysis of predictive values for patients undergoing RP

	OR	95% CI	*P*-value
Age	0.653	0.571–0.747	<0.001
BMI	1.077	0.975–1.189	0.144
ECOG PS			
0	Reference		
≥1	0.066	0.030–0.146	<0.001
Initial PSA	0.943	0.917–0.970	<0.001
Gleason score	1.029	0.736–1.439	0.866
Clinical T stage			
≤T2	Reference		
≥T3	1.029	0.490–2.164	0.939
Clinical N or M stage			
No	Reference		
Yes	0.316	0.078–1.279	0.106
Number of positive cores	1.069	0.943–1.213	0.297
Max percent of positive core	0.991	0.979–1.003	0.147

BMI, body mass index; ECOG PS, Eastern Cooperative Oncology Group Performance Status; M: metastasis; N, lymph nodes; PSA, prostate-specific antigen; T, tumor.

The median age of patients who underwent secondary treatment (n = 142) for prostate cancer was 77.0 (75.0–93.0) years and the median PSA was 34.0 (3.4–4656.0) ng/ml; 76.0% of patients who underwent secondary treatment had GSs ≥8, and 34.5% of patients had metastatic prostate cancer. The most common secondary treatment was RT (28.9%) followed by ADT (25.4%) and chemotherapy (23.2%) (Table S1).

The median age of patients who underwent tertiary treatment (n = 22) for prostate cancer was 77.5 (75.0–85.0) years and the median PSA was 146.2 (7.8–2380.0) ng/mL; 90.9% of patients who underwent tertiary treatment had GSs ≥8 and 59.1% had metastatic prostate cancer. The most common tertiary treatment was enzalutamide (36.4%) followed by abiraterone (18.2%) and docetaxel (18.2%) (Table S2).

Multivariable logistic regression analysis revealed that age (OR 0.900, *P* = 0.003), ECOG PS ≥ 2 versus 0 (OR 0.474, *P* = 0.015), GS (OR 1.461, *P* = 0.002), max percent of positive cores (OR 1.012, *P* = 0.031), and clinical stage N1 or M1 (OR 2.276, *P* = 0.003) predicted secondary treatment. No other predicting factor for undergoing secondary treatment were detected (Table S3).

**Fig. 3:**
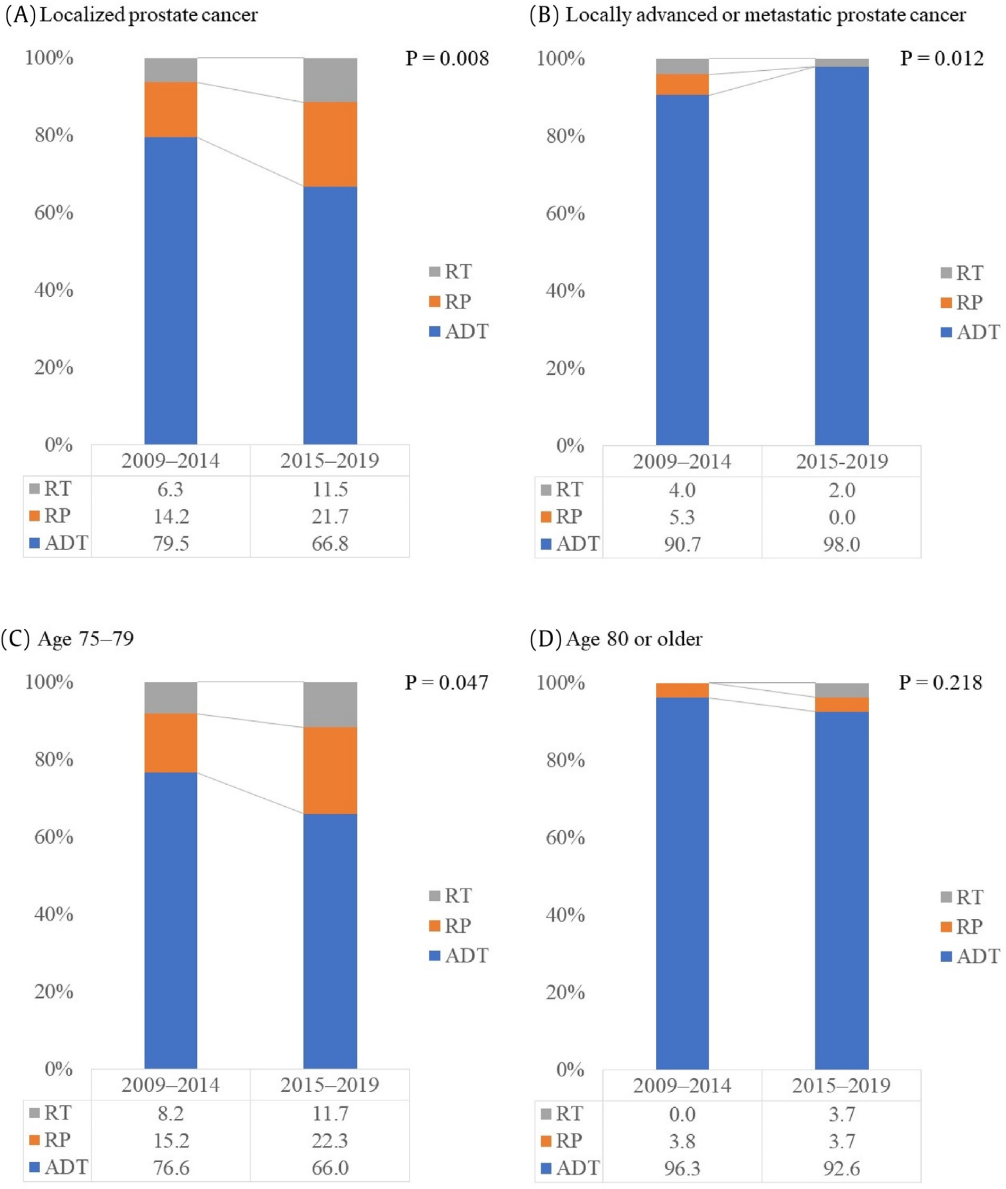
Comparisons of primary treatment patterns between 2009–2014 and 2015–2019 according to age and clinical stage group.

Compared to baseline data, clinicopathological characteristics and primary treatment patterns in 2009–2014 and 2015–2019 according to age and clinical stage group, localized prostate cancer, and the age 75–79 group in 2015–2019 had a significantly lower ratio of ADT and a higher ratio of RP and RT as the primary treatments than in 2009–2014 (Fig. 3). The ratio of ECOG PS 0 in 2015–2019 was significantly higher in localized prostate cancer and the 75–79 age group (Table S4).

## 4 Discussion

In the present study, the most common treatment option for prostate cancer in patients over 75 years was MAB. In previous studies, most patients over 75 years underwent ADT for prostate cancer, and the ratio of patients who underwent RP was lower in older patients than the ratio in younger ages.^8^^,^^9^

Younger age and lower ECOG PS and serum PSA levels predicted primary treatment of prostate cancer with RP in this study. Moreover, localized prostate cancer and the 75–79 age group in 2015–2019 had significantly higher ratios of ECOG PS 0 and RP as the primary treatments than those in 2009–2014. RP is indicated for clinically localized patients with prostate cancer and with a life expectancy of 10 years or more.^6^ The ratio of localized prostate cancer in patients over 75 years in the present study was similar to the ratio of localized prostate cancer in the Korea Central Cancer Registry (KCCR) annual report (56.0% vs. 53.0%). However, the ratio of metastatic prostate cancer in patients over 75 years in our study was higher than the ratio of metastatic prostate cancer in the KCCR annual report (19.2% vs. 10.1%).^4^ Increased ECOG PS is associated with shorter median survival periods in patients with advanced cancer .^12^ Shorter life expectancy due to older age and/or increased ECOG PS and a higher rate of metastatic prostate cancer may explain the lower rate of RP in patients with prostate cancer and 75 years or older.^4^^,^^6^^,^^12^

The present study showed that the proportion of RP increased from 11.6% in 2009–2014 to 15.8% in 2015–2019, and the proportion of ADP decreased from 82.9% in 2009–2014 to 75.3% in 2015–2019. Several recent studies showed that the relative portion of patients with prostate cancer undergoing surgery, especially RARP, rapidly increased, and the portion of patients undergoing ADT decreased slightly from 2003 to 2014.^8^^,^^9^

Life expectancy is continuously increasing; the age with a 10-year life expectancy increased from 75 years in 2009 to 77 years in 2019.^3^ Although the proportions of localized and regional prostate cancers did not dramatically increase from 2009 to 2019 (75.7% vs. 78.5%), the proportion of patients with prostate cancer and aged 75 years or older increased (27.4% vs. 35.9%).^13^^,^^14^

In the present study, the ratios of patients who underwent RP in the clinical stage T3 or more was 13.8% in 2009–2014 and 23.5% in 2015–2019. However, the ratio of patients who underwent RP in the clinical N or M stage did not increase. A retrospective study that evaluated the changing trend of RARP also reported that patients who underwent RARP from 2013 to 2019 had more unfavorable disease characteristics.^15^ Recently, the indications for RP were extended from localized or regional prostate cancer to node-positive or metastatic prostate cancer.^16^, ^17^, ^18^ In addition, the role of cytoreductive RP for metastatic prostate cancer is being investigated in several clinical trials.^19^

Several studies compared complications, functional outcomes, and oncological outcomes in patients who underwent RARP versus RRP.^20^, ^21^, ^22^, ^23^ Two meta-analyses demonstrated that RARP had lower complication rates and better functional outcomes than RRP.^20^^,^^21^ One meta-analysis reported that biochemical recurrence was lower in patients with RARP, but recurrence-free survival was similar between the two groups.^21^ A prospective, multicenter, controlled, non-randomized trial comparing RARP with RRP reported lower erectile dysfunction, positive surgical margins, biochemical recurrence, and prostate cancer-specific mortality for RARP.^22^ However, a randomized clinical phase 3 study comparing RARP with RRP reported similar functional outcomes between the two groups and lower biochemical recurrence at 24 months.^23^ The increasing life expectancy, the expansion of indications for surgical treatment, and the advantages of RARP may have increased the portion of prostate cancer patients aged 75 years or over undergoing surgical treatment, especially RARP.^3^^,^^5^^,^^8^^,^^14^^,^^16^^,^^17^^,^^19^, ^20^, ^21^, ^22^, ^23^

Among MAB regimens, leuprolide + bicalutamide was the most common in the present study. The most common luteinizing hormone-releasing hormone agonist for combination and monotherapy was leuprolide (57.8%), followed by goserelin (21.8%) and triptorelin (7.7%). A multi-institutional, prospective, observational study about hormone treatment for prostate cancer patients in Korea reported similar results.^24^ Although the authors reported that the efficacy of leuprorelin was lower than other luteinizing hormone-releasing hormone agonists, leuprorelin was the most common hormone therapy for prostate cancer; the authors hypothesized that this trend was due to cost-effectiveness.^24^

This study had several limitations. First, this study was retrospective, which may cause selection bias. Second, patients in this study came from only five institutions in Korea and may not reflect all prostate cancer patients in Korea. Last, this study contained clinical stage and biopsy data, but survival outcomes were not included. Thus, we could not estimate survival outcomes. Further studies, including survival outcome studies with more patients, are warranted.

In conclusion, the most used treatment option in patients with prostate cancer over 75 years was MAB. Among MAB regimens, leuprolide + bicalutamide was the most common. Age, ECOG PS, and PSA may be the useful indicators of surgical treatment, and the proportion of patients who underwent surgical treatment increased. Younger patients with high GSs and advanced clinical stages are more likely to receive secondary treatment.

## Conflict of interest

The authors have nothing to disclose.

## References

[1] Cho S.T., Na H.R. (2022). Urology and geriatrics in Korea: present status and future directions. Int Neurourol J.

[2] Population, Households and Housing Units [Internet]. KOSIS; [cited 2022 June 01]. Available from: https://kosis.kr/statHtml/statHtml.do?orgId=101&tblId=DT_1IN1502&vw_cd=MT_ETITLE&list_id=A11_2015_1&scrId=&language=en&seqNo=&lang_mode=en&obj_var_id=&itm_id=&conn_path=MT_ETITLE&path=%252Feng%252FstatisticsList%252FstatisticsListIndex.do.

[3] Complete life tables [Internet]. KOSIS; [cited 2022 June 01]. Available from: http://kosis.kr/eng/statisticsList/statisticsListIndex.do?menuId=M_01_01&vwcd=MT_ETITLE&parmTabId=M_01_01&parentId=F.1;F_29.2;#SelectStatsBoxDiv.

[4] Kang M.J., Won Y.J., Lee J.J., Jung K.W., Kim H.J., Kong H.J. (2022). Cancer statistics in Korea: incidence, mortality, survival, and prevalence in 2019. Cancer Res Treat.

[5] Korea Central Cancer Registry, National Cancer Center (2021).

[6] Prostate Cancer (Version 3. 2022) [Internet]. National Comprehensive Cancer Network; [cited 2022 May 03]. Available from: https://www.nccn.org/professionals/physician_gls/pdf/prostate.pdf.

[7] Bratt O., Folkvaljon Y., Hjalm Eriksson M., Akre O., Carlsson S., Drevin L. (2015). Undertreatment of men in their seventies with high-risk nonmetastatic prostate cancer. Eur Urol.

[8] Kang H.W., Yun S.J., Chung J.I., Choi H., Kim J.H., Yu H.S. (2019). National practice patterns and direct medical costs for prostate cancer in Korea across a 10 year period: a nationwide population-based study using a national health insurance database. BMC Health Serv Res.

[9] Park J., Suh B., Shin D.W., Hong J.H., Ahn H. (2016). Changing patterns of primary treatment in Korean men with prostate cancer over 10 years: a nationwide population based study. Cancer Res Treat.

[10] Ryu J.H., Kim Y.B., Jung T.Y., Kim S.I., Byun S.S., Kwon D.D. (2016). Radical Prostatectomy in Korean men aged 75-years or older: safety and efficacy in comparison with patients aged 65-69 Years. J Kor Med Sci.

[11] Ko Y.H. (2021). The comparison of the survival outcome between robotic-assisted radical prostatectomy and radiation therapy for localized prostate cancer in men over 70 years: Korean Nationwide Observational Study. J Robot Surg.

[12] Jang R.W., Caraiscos V.B., Swami N., Banerjee S., Mak E., Kaya E. (2014). Simple prognostic model for patients with advanced cancer based on performance status. J Oncol Pract.

[13] Korea Central Cancer Registry, National Cancer Center (2018).

[14] Korea Central Cancer Registry, National Cancer Center (2011).

[15] Garg H., Seth A., Singh P., Kumar R. (2021). Changing trends in robot-assisted radical prostatectomy: Inverse stage migration-A retrospective analysis. Prostate Int.

[16] Costello A.J. (2020). Considering the role of radical prostatectomy in 21st century prostate cancer care. Nat Rev Urol.

[17] Veeratterapillay R., Goonewardene S.S., Barclay J., Persad R., Bach C. (2017). Radical prostatectomy for locally advanced and metastatic prostate cancer. Ann R Coll Surg Engl.

[18] Jenjitranant P., Touijer K.A. (2019). Role of surgery in oligometastatic prostate cancer. Prostate Int.

[19] Ranasinghe W., Chapin B.F., Kim I.Y., Sooriakumaran P., Lawrentschuk N. (2020). The cytoreductive prostatectomy in metastatic prostate cancer: what the individual trials are hoping to answer. BJU Int.

[20] Du Y., Long Q., Guan B., Mu L., Tian J., Jiang Y. (2018). Robot-assisted radical prostatectomy is more beneficial for prostate cancer patients: a system review and meta-analysis. Med Sci Mon Int Med J Exp Clin Res.

[21] Seo H.J., Lee N.R., Son S.K., Kim D.K., Rha K.H., Lee S.H. (2016). Comparison of robot-assisted radical prostatectomy and open radical prostatectomy outcomes: a systematic review and meta-analysis. Yonsei Med J.

[22] Lantz A., Bock D., Akre O., Angenete E., Bjartell A., Carlsson S. (2021). Functional and oncological outcomes after open versus robot-assisted laparoscopic radical prostatectomy for localised prostate cancer: 8-year follow-up. Eur Urol.

[23] Coughlin G.D., Yaxley J.W., Chambers S.K., Occhipinti S., Samaratunga H., Zajdlewicz L. (2018). Robot-assisted laparoscopic prostatectomy versus open radical retropubic prostatectomy: 24-month outcomes from a randomised controlled study. Lancet Oncol.

[24] Kim J.K., Kim J.J., Gang T.W., Kwon T.K., Kim H.S., Park S.C. (2019). The current status of hormone treatment for prostate cancer patients in Korean real-world practice: a multi-institutional observational study. Asian J Androl.

