# Supplementary material for: Current treatment patterns within 1 year after prostate cancer diagnosis in Korean patients over 75 years old: a retrospective multicenter study
Source: Prostate Int. 2022 Aug 29;11(1):34–9. doi: 10.1016/j.prnil.2022.08.003 (PMC9995689; doi:10.1016/j.prnil.2022.08.003)
Supplement: Multimedia component 1 [file mmc1.docx]

| **Supplemental materials**  **Table S1. Baseline and clinicopathological characteristics of patients who underwent secondary treatment for prostate cancer** | | |
| --- | --- | --- |
| Characteristics | Patients (n = 142) |  |
| Age, years, median (range) | 77.0 (75.0–93.0) |  |
| BMI, kg/m^2^, mean ± SD | 23.0 ± 3.0 |  |
| ECOG PS, n (%) |  |  |
| 0 | 85 (59.9) |  |
| 1 | 35 (24.6) |  |
| 2 | 22 (15.5) |  |
| 3 | 0 (0.0) |  |
| Hypertension, n (%) | 67 (47.2) |  |
| Diabetes mellitus, n (%) | 28 (19.7) |  |
| First treatment choice, n (%) |  |  |
| ADT | 106 (74.6) |  |
| RT | 9 (6.3) |  |
| RP | 21 (19.8) |  |
| AS or WW | 2 (1.4) |  |
| Palliative TURP | 4 (2.8) |  |
| Second treatment choice, n (%) |  |  |
| RT | 41 (28.9) |  |
| ADT | 36 (25.4) |  |
| Chemotherapy (Docetaxel) | 20 (14.1) |  |
| Chemotherapy (Estramustine) | 12 (8.5) |  |
| Chemotherapy (Mithoxantrone) | 1 (0.7) |  |
| Enzalutamide | 9 (6.3) |  |
| Abiraterone | 10 (7.0) |  |
| RP | 8 (5.6) |  |
| WW | 5 (3.5) |  |
| Initial PSA, ng/ml, median (range) | 34.0 (3.4–4656.0) |  |
| Prostate volume, cc, median (range) | 38.4 (15.2–124.0) |  |
| Gleason score, n (%) |  |  |
| ≤6 | 5 (3.5) |  |
| 7 | 29 (20.4) |  |
| 8 | 46 (32.4) |  |
| 9 | 45 (31.7) |  |
| 10 | 17 (12.0) |  |
| Number of positive cores, median (range) | 8.0 (1.0–30.0) |  |
| Max percent of positive core, median (range) | 95.0 (5.9–100.0) |  |
| Clinical T stage, n (%) |  |  |
| ≤T2 | 64 (45.1) |  |
| ≥T3 | 78 (54.9) |  |
| Clinical N stage, n (%) |  |  |
| N0 | 91 (64.1) |  |
| N1 | 51 (35.9) |  |
| Clinical M stage, n (%) |  |  |
| M0 | 93 (65.5) |  |
| M1 | 49 (34.5) |  |

ADT, androgen deprivation therapy; AS, active surveillance; BMI, body mass index; ECOG PS, Eastern Cooperative Oncology Group Performance Status; M, metastasis; N, lymph nodes; PSA, prostate specific antigen; RP, radical prostatectomy; RT, radiation therapy; TUR, transurethral resection of the prostate; T, tumor; WW, watchful waiting

| **Table S2. Baseline and clinicopathological characteristics of patients who underwent tertiary treatment for prostate cancer** | | |
| --- | --- | --- |
| Characteristics | Patients (n = 22) |  |
| Age, years, median (range) | 77.5 (75.0–85.0) |  |
| BMI, kg/m^2^, mean ± SD | 22.0 ± 4.0 |  |
| ECOG PS, n (%) |  |  |
| 0 | 11 (50.0) |  |
| 1 | 4 (18.2) |  |
| 2 | 7 (31.8) |  |
| 3 | 0 (0.0) |  |
| Hypertension, n (%) | 9 (40.9) |  |
| Diabetes mellitus, n (%) | 1 (4.5) |  |
| First treatment choice |  |  |
| ADT | 21 (95.5) |  |
| RP | 1 (4.5) |  |
| Second treatment choice |  |  |
| Chemotherapy (Docetaxel) | 11 (50.0) |  |
| Chemotherapy (Estramustine) | 3 (13.6) |  |
| ADT | 3 (13.6) |  |
| RT | 3 (13.6) |  |
| RP | 1 (4.5) |  |
| WW | 1 (4.5) |  |
| Third treatment choice |  |  |
| Chemotherapy (Docetaxel) | 4 (18.2) |  |
| Chemotherapy (Estramustine) | 3 (13.6) |  |
| Enzalutamide | 8 (36.4) |  |
| Abiraterone | 4 (18.2) |  |
| ADT | 2 (9.1) |  |
| ADT + RT | 1 (4.5) |  |
| Initial PSA, ng/ml, median (range) | 146.2 (7.8–2380.0) |  |
| Prostate volume, cc, median (range) | 40.0 (17.0–86.0) |  |
| Gleason score, n (%) |  |  |
| 7 | 2 (9.1) |  |
| 8 | 5 (22.7) |  |
| 9 | 10 (45.5) |  |
| 10 | 5 (22.7) |  |
| Number of positive cores, median (range) | 12.0 (2.0–30.0) |  |
| Max percent of positive core, median (range) | 100.0 (20.0–100.0) |  |
| Clinical T stage, n (%) |  |  |
| ≤T2 | 4 (18.2) |  |
| ≥T3 | 18 (81.8) |  |
| Clinical N stage, n (%) |  |  |
| N0 | 6 (27.3) |  |
| N1 | 16 (72.7) |  |
| Clinical M stage, n (%) |  |  |
| M0 | 9 (40.9) |  |
| M1 | 13 (59.1) |  |

ADT, androgen deprivation therapy; BMI, body mass index; ECOG PS, Eastern Cooperative Oncology Group Performance Status; M, metastasis; N, lymph nodes; PSA, prostate specific antigen; RP, radical prostatectomy; RT, radiation therapy; T, tumor; WW, watchful waiting

| **Table S3. Analysis of predictive values for patients undergoing secondary treatment** | | | |
| --- | --- | --- | --- |
|  | OR | 95% CI | P value |
| Age | 0.900 | 0.841–0.964 | 0.003 |
| BMI | 0.963 | 0.896–1.034 | 0.299 |
| ECOG PS |  |  |  |
| 0 | Reference |  |  |
| 1 | 0.623 | 0.385–1.008 | 0.054 |
| ≥ 2 | 0.474 | 0.259–0.866 | 0.015 |
| Initial PSA | 1.000 | 1.000–1.001 | 0.240 |
| Gleason score | 1.461 | 1.156–1.847 | 0.002 |
| Clinical T stage |  |  |  |
| ≤T2 | Reference |  |  |
| ≥T3 | 0.694 | 0.413–1.166 | 0.168 |
| Clinical N or M stage |  |  |  |
| No | Reference |  |  |
| Yes | 2.276 | 1.322–3.920 | 0.003 |
| Number of positive cores | 0.980 | 0.907–1.060 | 0.615 |
| Max percent of positive core | 1.012 | 1.001–1.022 | 0.031 |

BMI, body mass index; ECOG PS, Eastern Cooperative Oncology Group Performance Status; M, metastasis; N, lymph nodes; PSA, prostate specific antigen; T, tumor

| **Table S4. Comparisons of baseline, clinicopathological characteristics, and primary treatment patterns between 2009–2014 and 2015–2019 according to age and clinical stage group.**  (A) Localized prostate cancer & age 75–79 years   \|  \| 2009–2014 (n = 122) \| 2015–2019 (n = 265) \| P-value \| \| --- \| --- \| --- \| --- \| \| Age, years \| 76.0 (75.0–79.0) \| 77.0 (75.0–79.0) \| 0.131 \| \| BMI, kg/m^2^ \| 23.0 ± 2.6 \| 23.5 ± 2.8 \| 0.100 \| \| ECOG PS \|  \|  \| 0.002 \| \| 0 \| 59 (48.8) \| 174 (65.7) \|  \| \| ≥1 \| 62 (51.2) \| 91 (34.3) \|  \| \| Hypertension \| 45 (37.5) \| 134 (50.6) \| 0.017 \| \| Diabetes mellitus \| 24 (20.0) \| 67 (25.3) \| 0.258 \| \| Initial PSA, ng/ml \| 11.6 (3.1–124.0) \| 10.4 (1.8–2357.7) \| 0.555 \| \| Total prostate volume, cc \| 36.0 (6.5–175.0) \| 35.0 (10.0–124.0) \| 0.824 \| \| Gleason score \|  \|  \| 0.501 \| \| ≤6 \| 24 (19.7) \| 50 (18.9) \|  \| \| 7 \| 49 (40.2) \| 96 (36.2) \|  \| \| ≥8 \| 49 (40.2) \| 119 (44.9) \|  \| \| Number of positive cores \| 5.0 (1.0–13.0) \| 5.0 (1.0–13.0) \| 0.630 \| \| Max percent of positive core, % \| 66.5 (1.0–100.0) \| 60.0 (4.8–100.0) \| 0.479 \| \| Clinical T stage \|  \|  \| 0.014 \| \| ≤T2 \| 98 (80.3) \| 181 (68.3) \|  \| \| ≥T3 \| 24 (19.7) \| 84 (31.7) \|  \| \| Clinical N & M stage \|  \|  \|  \| \| N0M0 \| 122 (100.0) \| 265 (100.0) \|  \| \| N1 or M1 \| 0 (0.0) \| 0 (0.0) \|  \| \| Primary treatment pattern \|  \|  \| 0.018 \| \| ADT \| 87 (71.3) \| 149 (56.2) \|  \| \| RP \| 24 (19.7) \| 78 (29.4) \|  \| \| RT \| 11 (9.0) \| 38 (14.3) \|  \| \| Primary treatment pattern \|  \|  \| 0.005 \| \| ADT \| 87 (71.3) \| 149 (56.2) \|  \| \| RP & RT \| 35 (28.7) \| 116 (43.8) \|  \|   ADT, androgen deprivation therapy; BMI, body mass index; ECOG PS, Eastern Cooperative Oncology Group Performance Status; M, metastasis; N, lymph nodes; PSA, prostate specific antigen; RP, radical prostatectomy; T, tumor.  (B) Localized prostate cancer and aged 80 years or older | | | |
| --- | --- | --- | --- | --- | --- | --- | --- | --- | --- | --- | --- | --- | --- | --- | --- | --- | --- | --- | --- | --- | --- | --- | --- | --- | --- | --- | --- | --- | --- | --- | --- | --- | --- | --- | --- | --- | --- | --- | --- | --- | --- | --- | --- | --- | --- | --- | --- | --- | --- | --- | --- | --- | --- | --- | --- | --- | --- | --- | --- | --- | --- | --- | --- | --- | --- | --- | --- | --- | --- | --- | --- | --- | --- | --- | --- | --- | --- | --- | --- | --- | --- | --- | --- | --- | --- | --- | --- | --- | --- | --- | --- | --- | --- | --- | --- | --- | --- | --- | --- | --- | --- | --- | --- | --- | --- | --- | --- | --- | --- | --- | --- | --- | --- | --- | --- | --- | --- | --- | --- |
|  | 2009–2014 (n = 54) | 2015–2019 (n = 127) | P-value |
| Age, years | 81.0 (80.0–88.0) | 81.0 (80.0–93.0) | 0.017 |
| BMI, kg/m^2^ | 22.5 ± 2.2 | 23.0 ± 3.1 | 0.375 |
| ECOG PS |  |  | 0.033 |
| 0 | 23 (42.6) | 76 (59.8) |  |
| ≥1 | 31 (57.4) | 51 (40.2) |  |
| Hypertension | 26 (48.1) | 62 (48.8) | 0.934 |
| Diabetes mellitus | 10 (18.5) | 28 (22.0) | 0.594 |
| Initial PSA, ng/ml | 29.0 (3.6–263.7) | 14.5 (2.5–428.9) | 0.007 |
| Total prostate volume, cc | 35.0 (19.0–215.8) | 36.0 (15.0–141.0) | 0.246 |
| Gleason score |  |  | 0.034 |
| ≤6 | 2 (3.7) | 24 (18.9) |  |
| 7 | 19 (35.2) | 38 (29.9) |  |
| ≥8 | 33 (61.1) | 65 (51.2) |  |
| Number of positive cores | 7.0 (1.0–13.0) | 6.0 (1.0–12.0) | 0.239 |
| Max percent of positive core, % | 83.3 (1.0–100.0) | 68.5 (5.0–100.0) | 0.088 |
| Clinical T stage |  |  | 0.026 |
| ≤T2 | 29 (53.7) | 90 (70.9) |  |
| ≥T3 | 25 (46.3) | 37 (29.1) |  |
| Clinical N & M stage |  |  |  |
| N0M0 | 54 (100.0) | 127 (100.0) |  |
| N1 or M1 | 0 (0.0) | 0 (0.0) |  |
| Primary treatment pattern |  |  | 0.107 |
| ADT | 53 (98.1) | 113 (89.0) |  |
| RP | 1 (1.9) | 7 (5.5) |  |
| RT | 0 (0.0) | 7 (5.5) |  |
| Primary treatment pattern |  |  | 0.042 |
| ADT | 53 (98.1) | 113 (89.0) |  |
| RP & RT | 1 (1.9) | 14 (11.0) |  |

ADT, androgen deprivation therapy; BMI, body mass index; ECOG PS, Eastern Cooperative Oncology Group Performance Status; M, metastasis; N, lymph nodes; PSA, prostate specific antigen; RP, radical prostatectomy; T, tumor.

(C) Locally advanced or metastatic prostate cancer and the 75–79 age group

|  | 2009–2014 (n = 49) | 2015–2019 (n = 85) | *P*-value |
| --- | --- | --- | --- |
| Age, years | 77.0 (75.0–79.0) | 77.0 (75.0–79.0) | 0.932 |
| BMI, kg/m^2^ | 23.0 ± 3.1 | 23.6 ± 2.8 | 0.192 |
| ECOG PS |  |  | 0.337 |
| 0 | 22 (44.9) | 31 (36.5) |  |
| ≥1 | 27 (55.1) | 54 (63.5) |  |
| Hypertension | 20 (40.8) | 41 (48.2) | 0.406 |
| Diabetes mellitus | 7 (14.3) | 21 (24.7) | 0.153 |
| Initial PSA, ng/ml | 100.0 (4.3–4852.9) | 98.0 (4.4–4823.0) | 0.430 |
| Total prostate volume, cc | 50.2 (14.0–194.0) | 14.0 (14.0–128.0) | 0.147 |
| Gleason score |  |  | 0.671 |
| ≤6 | 3 (6.1) | 2 (2.4) |  |
| 7 | 2 (4.1) | 7 (8.2) |  |
| ≥8 | 44 (89.8) | 76 (89.4) |  |
| Number of positive cores | 11.0 (1.0–18.0) | 11.0 (1.0–12.0) | 0.326 |
| Max percent of positive core, % | 100.0 (14.7–100.0) | 100.0 (20.0–100.0) | 0.947 |
| Clinical T stage |  |  | 0.073 |
| ≤T2 | 10 (20.4) | 8 (9.4) |  |
| ≥T3 | 39 (79.6) | 77 (90.6) |  |
| Clinical N & M stage |  |  |  |
| N0M0 | 0 (0.0) | 0 (0.0) |  |
| N1 or M1 | 49 (100.0) | 85 (100.0) |  |
| Primary treatment pattern |  |  | 0.130 |
| ADT | 44 (89.8) | 82 (96.5) |  |
| RP | 2 (4.1) | 0 (0.0) |  |
| RT | 3 (6.1) | 3 (3.5) |  |
| Primary treatment pattern |  |  | 0.141 |
| ADT | 44 (89.8) | 82 (96.5) |  |
| RP & RT | 5 (10.2) | 3 (3.5) |  |

ADT, androgen deprivation therapy; BMI, body mass index; ECOG PS, Eastern Cooperative Oncology Group Performance Status; M, metastasis; N, lymph nodes; PSA, prostate specific antigen; RP, radical prostatectomy; T, tumor.

(D) Locally advanced or metastatic prostate cancer and aged 80 years or older

|  | 2009–2014 (n = 26) | 2015–2019 (n = 62) | *P*-value |
| --- | --- | --- | --- |
| Age, years | 81.0 (80.0–90.0) | 83.0 (80.0–94.0) | 0.048 |
| BMI, kg/m^2^ | 22.2 ± 2.2 | 22.9 ± 3.4 | 0.322 |
| ECOG PS |  |  | 0.296 |
| 0 | 9 (34.6) | 29 (46.8) |  |
| ≥1 | 17 (65.4) | 33 (53.2) |  |
| Hypertension | 11 (44.0) | 27 (43.5) | 0.969 |
| Diabetes mellitus | 5 (20.5) | 6 (9.7) | 0.190 |
| Initial PSA, ng/ml | 67.2 (6.2–6520.0) | 100.0 (7.8–4656.0) | 0.152 |
| Total prostate volume, cc | 39.0 (13.0–82.0) | 42.0 (15.0–224.6) | 0.834 |
| Gleason score |  |  | 0.193 |
| ≤6 | 0 (0.0) | 1 (1.6) |  |
| 7 | 6 (23.1) | 5 (8.1) |  |
| ≥8 | 20 (76.9) | 56 (90.3) |  |
| Number of positive cores | 10.0 (2.0–12.0) | 10.0 (3.0–12.0) | 0.675 |
| Max percent of positive core, % | 100.0 (20.0–100.0) | 100.0 (10.0–100.0) | 0.147 |
| Clinical T stage |  |  | 0.333 |
| ≤T2 | 7 (26.9) | 11 (17.7) |  |
| ≥T3 | 19 (73.1) | 51 (82.3) |  |
| Clinical N & M stage |  |  |  |
| N0M0 | 0 (0.0) | 0 (0.0) |  |
| N1 or M1 | 26 (100.0) | 62 (100.0) |  |
| Primary treatment pattern |  |  | 0.027 |
| ADT | 24 (92.3) | 62 (100.0) |  |
| RP | 2 (7.7) | 0 (0.0) |  |
| RT | 0 (0.0) | 0 (0.0) |  |
| Primary treatment pattern |  |  | 0.085 |
| ADT | 24 (92.3) | 62 (100.0) |  |
| RP & RT | 2 (7.7) | 0 (0.0) |  |

ADT, androgen deprivation therapy; BMI, body mass index; ECOG PS, Eastern Cooperative Oncology Group Performance Status; M, metastasis; N, lymph nodes; PSA, prostate specific antigen; RP, radical prostatectomy; T, tumor.

(E) Localized cancer

|  | 2009–2014 (n = 176) | 2015–2019 (n = 392) | P-value |
| --- | --- | --- | --- |
| Age, years | 78.0 (75.0–88.0) | 78.0 (75.0–93.0) | 0.112 |
| BMI, kg/m^2^ | 22.9 ± 2.5 | 23.3 ± 2.9 | 0.056 |
| ECOG PS |  |  | <0.001 |
| 0 | 82 (46.9) | 250 (63.8) |  |
| ≥1 | 93 (53.1) | 142 (36.2) |  |
| Hypertension | 71 (40.8) | 196 (50.0) | 0.043 |
| Diabetes mellitus | 34 (19.5) | 95 (24.2) | 0.219 |
| Initial PSA, ng/ml | 14.4 (3.1–263.7) | 11.3 (1.8–2357.7) | 0.061 |
| Total prostate volume, cc | 35.5 (6.5–215.8) | 35.0 (10.0–141.0) | 0.662 |
| Gleason score |  |  | 0.580 |
| ≤6 | 26 (14.8) | 73 (18.9) |  |
| 7 | 68 (38.6) | 134 (34.2) |  |
| ≥8 | 82 (46.6) | 184 (46.9) |  |
| Number of positive cores | 5.0 (1.0–30.0) | 5.0 (1.0–13.0) | 0.310 |
| Max percent of positive core, % | 71.0 (1.0–100.0) | 60.0 (4.8–100.0) | 0.152 |
| Clinical T stage |  |  | 0.467 |
| ≤T2 | 127 (72.2) | 271 (69.1) |  |
| ≥T3 | 49 (27.8) | 121 (30.9) |  |
| Clinical N & M stage |  |  |  |
| N0M0 | 176 (100.0) | 392 (100.0) |  |
| N1 or M1 | 0 (0.0) | 0 (0.0) |  |
| Primary treatment pattern |  |  | 0.008 |
| ADT | 140 (79.5) | 262 (66.8) |  |
| RP | 25 (14.2) | 85 (21.7) |  |
| RT | 11 (6.3) | 45 (11.5) |  |
| Primary treatment pattern |  |  | 0.002 |
| ADT | 140 (79.5) | 262 (66.8) |  |
| RP & RT | 36 (20.5) | 130 (33.2) |  |

ADT, androgen deprivation therapy; BMI, body mass index; ECOG PS, Eastern Cooperative Oncology Group Performance Status; M, metastasis; N, lymph nodes; PSA, prostate specific antigen; RP, radical prostatectomy; T, tumor.

(F) Locally advanced or metastatic prostate cancer

|  | 2009–2014 (n = 75) | 2015–2019 (n = 147) | *P*-value |
| --- | --- | --- | --- |
| Age, years | 78.0 (75.0–90.0) | 79.0 (75.0–94.0) | 0.151 |
| BMI, kg/m^2^ | 22.7 ± 2.7 | 23.3 ± 3.1 | 0.154 |
| ECOG PS |  |  | 0.941 |
| 0 | 31 (41.3) | 60 (40.8) |  |
| ≥1 | 44 (58.7) | 87 (59.2) |  |
| Hypertension | 31 (41.9) | 68 (46.3) | 0.538 |
| Diabetes mellitus | 12 (16.2) | 27 (18.4) | 0.692 |
| Initial PSA, ng/ml | 100.0 (4.2–6520.0) | 99.0 (4.4–4823.0) | 0.858 |
| Total prostate volume, cc | 48.3 (13.0–194.0) | 41.8 (14.0–224.6) | 0.295 |
| Gleason score |  |  | 0.284 |
| ≤6 | 3 (4.0) | 3 (2.0) |  |
| 7 | 8 (10.7) | 12 (8.2) |  |
| ≥8 | 64 (85.3) | 132 (89.8) |  |
| Number of positive cores | 11.0 (1.0–18.0) | 11.0 (1.0–12.0) | 0.557 |
| Max percent of positive core, % | 100.0 (14.7–100.0) | 100.0 (10.0–100.0) | 0.402 |
| Clinical T stage |  |  | 0.063 |
| ≤T2 | 17 (22.7) | 19 (12.9) |  |
| ≥T3 | 58 (77.3) | 128 (87.1) |  |
| Clinical N & M stage |  |  |  |
| N0M0 | 0 (0.0) | 0 (0.0) |  |
| N1 or M1 | 75 (100.0) | 147 (100.0) |  |
| Primary treatment pattern |  |  | 0.012 |
| ADT | 68 (90.7) | 144 (98.0) |  |
| RP | 4 (5.3) | 0 (0.0) |  |
| RT | 3 (4.0) | 3 (2.0) |  |
| Primary treatment pattern |  |  | 0.033 |
| ADT | 68 (90.7) | 144 (98.0) |  |
| RP & RT | 7 (9.3) | 3 (2.0) |  |

ADT, androgen deprivation therapy; BMI, body mass index; ECOG PS, Eastern Cooperative Oncology Group Performance Status; M, metastasis; N, lymph nodes; PSA, prostate specific antigen; RP, radical prostatectomy; T, tumor.

(G) Aged 75–79 years

|  | 2009–2014 (n = 171) | 2015–2019 (n = 350) | *P*-value |
| --- | --- | --- | --- |
| Age, years | 77.0 (75.0–79.0) | 77.0 (75.0–79.0) | 0.171 |
| BMI, kg/m^2^ | 23.0 ± 2.6 | 23.6 ± 2.8 | 0.037 |
| ECOG PS |  |  | 0.019 |
| 0 | 81 (47.6) | 205 (58.6) |  |
| ≥1 | 89 (52.4) | 145 (41.4) |  |
| Hypertension | 65 (38.5) | 175 (50.0) | 0.013 |
| Diabetes mellitus | 31 (18.3) | 88 (25.1) | 0.084 |
| Initial PSA, ng/ml | 17.3 (3.1–4852.9) | 14.8 (1.8–4823.0) | 0.260 |
| Total prostate volume, cc | 37.9 (6.5–194.0) | 36.0 (10.0–128.0) | 0.418 |
| Gleason score |  |  | 0.108 |
| ≤6 | 27 (15.8) | 52 (14.9) |  |
| 7 | 51 (29.8) | 103 (29.4) |  |
| ≥8 | 93 (54.4) | 195 (55.7) |  |
| Number of positive cores | 6.0 (1.0–18.0) | 6.0 (1.0–13.0) | 0.234 |
| Max percent of positive core, % | 81.7 (1.0–100.0) | 72.2 (4.8–100.0) | 0.263 |
| Clinical T stage |  |  | 0.048 |
| ≤T2 | 108 (63.2) | 189 (54.0) |  |
| ≥T3 | 63 (36.8) | 161 (46.0) |  |
| Clinical N & M stage |  |  | 0.284 |
| N0M0 | 122 (71.3) | 265 (75.7) |  |
| N1 or M1 | 49 (28.7) | 85 (24.3) |  |
| Primary treatment pattern |  |  | 0.047 |
| ADT | 131 (76.6) | 231 (66.0) |  |
| RP | 26 (15.2) | 78 (22.3) |  |
| RT | 14 (8.2) | 41 (11.7) |  |
| Primary treatment pattern |  |  | 0.014 |
| ADT | 131 (76.6) | 231 (66.0) |  |
| RP & RT | 40 (23.4) | 119 (34.0) |  |

ADT, androgen deprivation therapy; BMI, body mass index; ECOG PS, Eastern Cooperative Oncology Group Performance Status; M, metastasis; N, lymph nodes; PSA, prostate specific antigen; RP, radical prostatectomy; T, tumor.

(H) Aged 80 years or older

|  | 2009–2014 (n = 80) | 2015–2019 (n = 189) | *P*-value |
| --- | --- | --- | --- |
| Age, years | 81.0 (80.0–90.0) | 82.0 (80.0–94.0) | 0.002 |
| BMI, kg/m^2^ | 22.4 ± 2.2 | 22.9 ± 3.2 | 0.197 |
| ECOG PS |  |  | 0.020 |
| 0 | 32 (40.0) | 105 (56.6) |  |
| ≥1 | 48 (60.0) | 84 (43.4) |  |
| Hypertension | 37 (46.8) | 89 (47.1) | 0.970 |
| Diabetes mellitus | 15 (19.0) | 34 (18.0) | 0.847 |
| Initial PSA, ng/ml | 40.0 (3.6–6520.0) | 25.3 (2.5–4656.0) | 0.201 |
| Total prostate volume, cc | 37.0 (13.0–215.8) | 37.0 (15.0–224.6) | 0.376 |
| Gleason score |  |  | 0.148 |
| ≤6 | 2 (2.5) | 25 (13.2) |  |
| 7 | 25 (31.3) | 43 (22.8) |  |
| ≥8 | 53 (66.3) | 121 (64.0) |  |
| Number of positive cores | 7.0 (1.0–13.0) | 7.0 (1.0–12.0) | 0.478 |
| Max percent of positive core, % | 91.5 (5.0–100.0) | 80.0 (5.0–100.0) | 0.052 |
| Clinical T stage |  |  | 0.206 |
| ≤T2 | 36 (45.0) | 101 (53.4) |  |
| ≥T3 | 44 (55.0) | 88 (46.6) |  |
| Clinical N & M stage |  |  | 0.961 |
| N0M0 | 54 (67.5) | 127 (67.2) |  |
| N1 or M1 | 26 (32.5) | 62 (32.8) |  |
| Primary treatment pattern |  |  | 0.218 |
| ADT | 77 (96.3) | 175 (92.6) |  |
| RP | 3 (3.8) | 7 (3.7) |  |
| RT | 0 (0.0) | 7 (3.7) |  |
| Primary treatment pattern |  |  | 0.411 |
| ADT | 77 (96.3) | 175 (92.6) |  |
| RP & RT | 3 (3.8) | 14 (7.4) |  |

ADT, androgen deprivation therapy; BMI, body mass index; ECOG PS, Eastern Cooperative Oncology Group Performance Status; M, metastasis; N, lymph nodes; PSA, prostate specific antigen; RP, radical prostatectomy; T, tumor.

(I) Total

|  | 2009–2014 (n = 251) | 2015–2019 (n = 539) | P-value |
| --- | --- | --- | --- |
| Age, years | 78.0 (75.0–90.0) | 78.0 (75.0–94.0) | 0.039 |
| BMI, kg/m^2^ | 22.8 ± 2.5 | 23.3 ± 3.0 | 0.015 |
| ECOG PS |  |  | 0.001 |
| 0 | 113 (45.2) | 310 (57.9) |  |
| ≥1 | 137 (54.8) | 229 (42.1) |  |
| Hypertension | 102 (41.1) | 264 (49.0) | 0.040 |
| Diabetes mellitus | 46 (18.5) | 122 (22.6) | 0.194 |
| Initial PSA, ng/ml | 21.4 (3.1–6520.0) | 18.0 (1.8–4823.0) | 0.112 |
| Total prostate volume, cc | 37.7 (6.5–215.8) | 36.4 (10.0–224.6) | 0.259 |
| Gleason score |  |  | 0.679 |
| ≤6 | 29 (11.6) | 77 (14.3) |  |
| 7 | 76 (30.3) | 146 (27.1) |  |
| ≥8 | 146 (58.2) | 316 (58.6) |  |
| Number of positive cores | 7.0 (1.0–30.0) | 6.0 (1.0–13.0) | 0.180 |
| Max percent of positive core, % | 90.0 (1.0–100.0) | 80.0 (4.9–100.0) | 0.059 |
| Clinical T stage |  |  | 0.348 |
| ≤T2 | 144 (57.4) | 290 (53.8) |  |
| ≥T3 | 107 (42.6) | 249 (46.2) |  |
| Clinical N & M stage |  |  | 0.448 |
| N0M0 | 176 (70.1) | 392 (72.7) |  |
| N1 or M1 | 75 (29.9) | 147 (27.3) |  |
| Primary treatment pattern |  |  | 0.056 |
| ADT | 208 (82.9) | 406 (75.3) |  |
| RP | 29 (11.6) | 85 (15.8) |  |
| RT | 14 (5.6) | 48 (8.9) |  |
| Primary treatment pattern |  |  | 0.018 |
| ADT | 208 (82.9) | 406 (75.3) |  |
| RP & RT | 43 (17.1) | 133 (24.7) |  |

ADT, androgen deprivation therapy; BMI, body mass index; ECOG PS, Eastern Cooperative Oncology Group Performance Status; M, metastasis; N, lymph nodes; PSA, prostate specific antigen; RP, radical prostatectomy; T, tumor.
